# Reshaping CAR-T cells through overexpression of T cell factor 1

**DOI:** 10.3389/fimmu.2025.1623869

**Published:** 2025-11-10

**Authors:** Hao Yao, Yuntian Ding, Qian Chen, Huixiu Han, David Sedloev, Carsten Müller-Tidow, Tim Sauer, Anita Schmitt, Michael Schmitt, Lei Wang

**Affiliations:** 1 Department of Internal Medicine V, University Clinic Heidelberg, Heidelberg University, Heidelberg, Germany; 2 Department of Nuclear Medicine, University Clinic Heidelberg, Heidelberg University, Heidelberg, Germany; 3 Department of Hematology, The Seventh Affiliated Hospital, Sun Yat-sen University, Shenzhen, China

**Keywords:** immunotherapy, CAR (chimeric antigen receptor) T cells, TCF (T-cell factor), CRS - cytokine release syndrome, T cell persistence

## Abstract

**Introduction:**

Although chimeric antigen receptor (CAR) T cell therapy has revolutionized treatment for hematologic malignancies, insufficient CAR-T cell persistence remains a major limitation. T cell factor 1 (TCF-1) is a transcription factor crucial for T cell development, self-renewal, and memory formation. However, CAR-T cells typically exhibit low TCF-1 expression. This study investigated whether restoring TCF-1 expression could enhance CAR-T cell persistence and functionality.

**Methods:**

Human peripheral blood T cells were transduced with third-generation CD19 or CD33 CAR retroviral vectors, with or without a TCF-1 (Tcf7.NGFR) construct. Phenotypic, functional, and transcriptional analyses were performed using flow cytometry, cytokine profiling, long-term killing assays, and RNA sequencing. Data mining and machine learning were applied for high-dimensional immunophenotyping.

**Results:**

TCF-1 overexpression generated CAR-T cells with reduced apoptosis, lower activation marker expression, and an increased proportion of naïve and stem cell–like subsets. These modified cells displayed a higher CD4⁺/CD8⁺ ratio, preserved proliferative capacity, and maintained cytotoxicity with attenuated cytokine release. Long-term co-culture assays demonstrated superior persistence and sustained tumor-killing activity in TCF-1–overexpressing CAR-T cells. Transcriptomic profiling revealed downregulation of apoptotic and cytokine release pathways, and enrichment of cell cycle and metabolic pathways supporting T cell longevity.

**Discussion:**

Overexpression of TCF-1 confers resistance to apoptosis, limits excessive activation, and promotes a less differentiated phenotype, collectively enhancing CAR-T cell persistence and long-term efficacy. These findings suggest that TCF-1 modulation represents a promising strategy to improve durability and safety of CAR-T cell therapy in relapsed or refractory hematologic malignancies.

## Introduction

Numerous studies have demonstrated the impressive efficacy of CAR-T cells targeting CD19 and BCMA in relapsed/refractory patients with hematological malignancies (1–8). Despite remarkable efficacy of CAR-T cells, disease progression occurs in a significant proportion of patients receiving CAR-T cell infusions, primarily due to insufficient expansion and persistence of the CAR-T cells (9, 10). The antitumor efficacy of CAR-T cells requires efficient expansion and persistence both *in vivo* and *in vitro (*11, 12).

T cell-specific transcription factor 1 (TCF-1) contains a single DNA-binding high mobility group (HMG) box. It plays a crucial role in the self-renewal of embryonic stem cells during mouse embryonic development (13). TCF-1 is essential for T cell development in the thymus and for the differentiation of mature T cells in peripheral tissues. It has the capability to regulate common transcription factors across different lineages to ensure appropriate lineage development (14). This is particularly significant for T cell differentiation, where TCF-1 drives similar gene circuits in multiple T cell subsets. For instance, TCF-1 induces Bcl6 expression to promote T follicular helper (Tfh), T follicular regulatory (Tfr), and T cell exhaustion-stem (Tex-stem) cells in mouse models (15). Additionally, TCF-1 can promote stem cell-like fates in CD4^+^ and CD8^+^ T cells in response to chronic stimulation. Recent studies have also demonstrated that TCF-1 is vital for the formation and maintenance of CD8^+^ T cell memory during acute infections, as well as for the regulation of CD8^+^ T cell exhaustion processes in chronic infections and tumors, influencing the differentiation, function, and maintenance of CD8^+^ exhausted T (Tex) cells (16). The TCF-1-centered transcriptional network plays a significant role in CD8^+^ T cells, providing numerous key targets for immunotherapy.

Although exhaustion can impair CAR-T cell activity, studies of chronic viral antigen exposure have identified a subset of CD8^+^ exhausted T cells (T_EX_) with memory-like features that support T cell persistence and function. These precursor exhausted T cells (T_PEX_) share characteristics with central memory T cells (T_CM_) but are transcriptionally and epigenetically distinct (17). TCF-1 is an essential transcription factor for the development and maintenance of both T_PEX_ and T_CM_.

The role of TCF-1 in CAR-T cell responses remains unknown. Therefore, in-depth investigation of the regulatory mechanisms of TCF-1 on CAR-T cells could be instrumental in developing enhanced immunotherapies for various diseases.

## Methods

### CAR-T cell and DT.CAR-T cell generation

Buffy coats from healthy donors were obtained from the German Red Cross Blood Donor Service (Baden-Württemberg, Mannheim, Germany). Peripheral blood mononuclear cells (PBMCs) were isolated and stimulated with anti-CD3/CD28 antibodies in the presence of IL-7 (10 ng/ml) and IL-15 (5 ng/ml). Activated T cells were transduced with a third-generation retroviral CAR construct (SFG.CD28/4-1BB/ζ) coding for CD19 or CD33. Dual-transduced CAR-T (DT.CAR-T) cells were generated using either CD19 or CD33 CAR along with a TCF-1 vector (SFG.Tcf7.NGFR). The culture medium was replenished every 2–3 days, and cells were analyzed on day 14.

### Long-term killing assay

CAR-T cells (0.25 × 10^5^) were co-cultured with tumor cells at effector-to-target (E:T) ratios of 1:1 or 1:2. Fresh tumor cells were added daily to challenge CAR-T cells until tumor growth was no longer controlled. Cells and supernatants were collected every two days for quantification, immunophenotyping, and cytokine profiling.

### Cell surface staining

A total of 5 × 10^5^ – 10 × 10^5^ cells were washed with 500 µL cold FACS buffer, pelleted, and resuspended in 100 µL FACS buffer. Cells were incubated with an antibody cocktail for 30 minutes in the dark at room temperature. After two washes with 1 mL FACS buffer and centrifugation at 500 g for 4 minutes, cell pellets were resuspended in 0.5 mL FACS buffer. Before flow cytometry, 5 µL of 7-AAD viability stain was added.

### Intracellular cytokine staining

CAR-T and DT.CAR-T cells were co-cultured with CD19^+^ tumor cells (Raji, Nalm-6) or CD33^+^ tumor cells (MV4-11) for five hours in the presence of monensin, brefeldin A, and CD107a antibody. Negative controls included CAR-T and DT.CAR-T cells cultured without tumor cells but with secretion inhibitors and CD107a antibody. Following incubation, cells were washed twice (500 g, 4 minutes), then stained for surface markers for 30 minutes. After fixation and permeabilization using the FoxP3 staining buffer, cells were stained for IFN-γ and TNF-α for another 30 minutes. Finally, cells were washed and resuspended in 500 µL FACS buffer before flow cytometry.

### Flow cytometry

Fluorochrome-conjugated antibodies were used for staining, with dead cells excluded using the LIVE/DEAD™ fixable near-infrared (IR) dead cell stain kit (Thermo Fisher) or 7AAD (BD Biosciences). CAR expression was identified using an anti-human goat F(ab)_2_ IgG (H+L) PE reagent (Dianova, Germany), and TCF-1 expression was detected via anti-human CD271 (NGFR) antibody. Fluorescence compensation was applied before acquisition, and appropriate fluorescence-minus-one (FMO) and non-transduced controls were included. Flow cytometric data were acquired using a BD LSR II™ flow cytometer (BD Biosciences) and analyzed with FlowJo™ v10 software.

## Results

### TCF-1 overexpressed CAR-T cells were successfully generated

**Figure 1:**
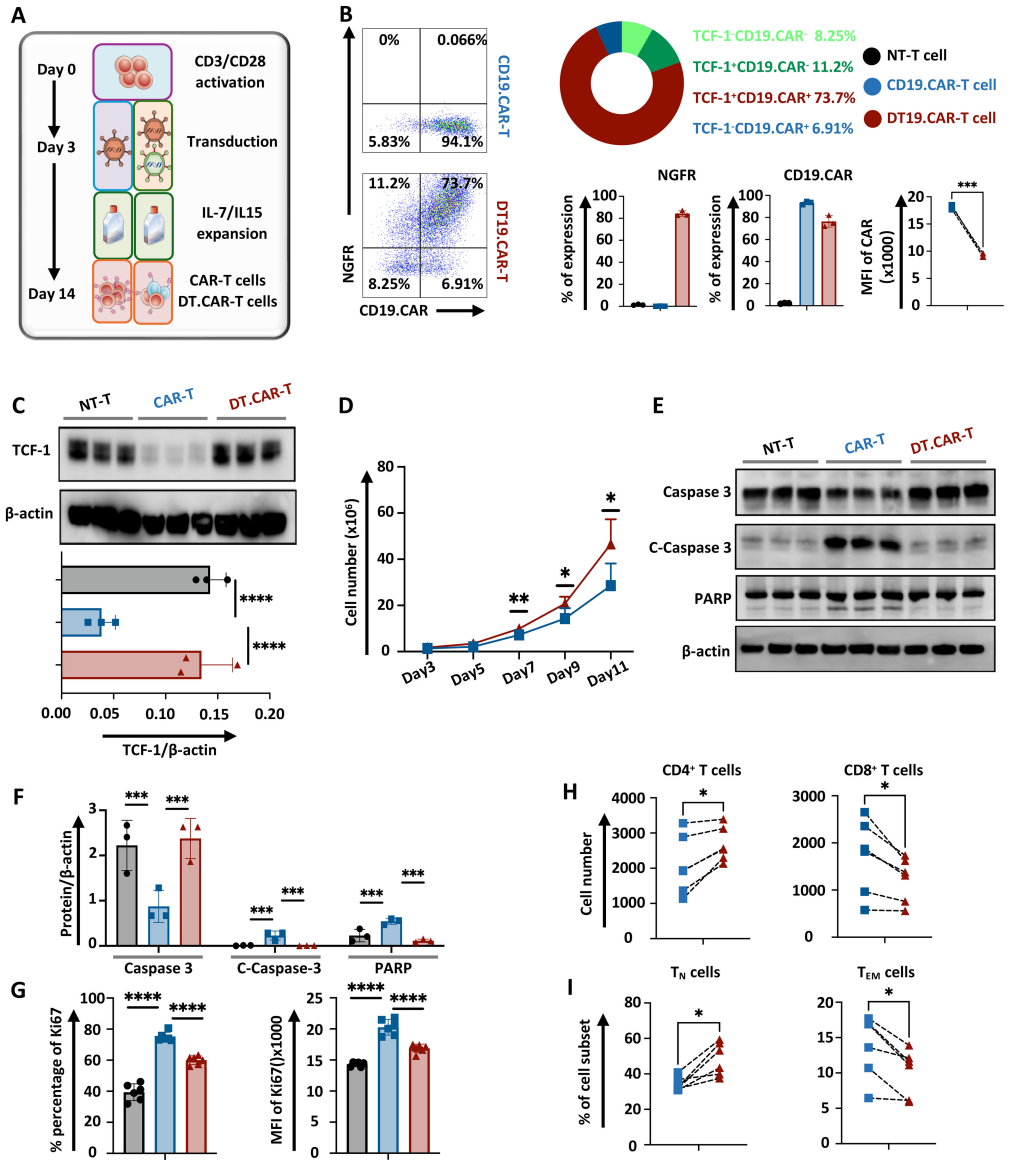
Effect of TCF-1 overexpression on CAR expression, cell expansion, apoptosis, cell component of CD19.CAR-T cells. **(A)** Representative figure of CD19.CAR-T cells and DT19.CAR-T cell generation (n=3). **(B)** Representative dolt plots of CD19.CAR and NGFR expression on CD19.CAR-T cells and DT19.CAR-T cells, and statistical analysis of CD19.CAR and NGFR expression on non-transduced T cells, CD19.CAR-T cells and DT19.CAR-T cells (n=3). **(C)** Statistical analysis protein level of TCF-1 on non-transduced T cells, CD19.CAR- T cells, and DT19.CAR-T cells, as detected by Western Blot (n=3). **(D)** Proliferation dynamics of CD19.CAR-T cells and DT19.CAR-T cells from Day 3 to Day 11 (n=6). **(E)** The expression of protein level of caspase 3, cleaved caspase 3 and PARP in non-transduced T cells, CD19.CAR-T cells and DT19.CAR-T cells (n=3). **(F)** Statistical analysis of the protein level of caspase 3, cleaved caspase3, and PARP in non-transduced T cells, CD19.CAR-T cells and DT19.CAR-T cells (n=3). **(G)** Expression of Ki67 on non-transduced T cells, CD19.CAR-T cells and DT19.CAR-T cells (n=6). **(H)** CD4/CD8 composition in CD19.CAR-T cells and DT19.CAR-T cells (n=6). **(I)** T cell subsets in CD19.CAR-T cells and DT19.CAR-T cells (n=6). A paired t-test was used for statistical analysis. (*P<0.05, **P<0.01, ***P<0.001, ****P<0.0001, ns= no significant difference).

In order to generate CAR-T cells which overexpress TCF-1, T cells were simultaneously co-transduced with viral vectors. In particular, a third-generation retroviral CD19.CAR vector (RV-SFG.CD19.CD28/4-1BB/ζ) was co-transduced together with an additional TCF-1 retroviral vector (RV-SFG.Tcf-7.NGFR) into T cells (**Figure 1A**). Both CD19.CAR-T cells and DT.CAR-T cells were successfully generated, demonstrating a consistent and stable CAR transduction efficiency (CD19.CAR-T cells: 93.20% ± 1.54, DT19.CAR-T cells: 76.30% ± 5.21). Of note, not only the frequency of CD19.CAR-T cells, but also the mean fluorescence intensity (MFI) of CD19.CAR was decreased in DT.CAR-T cells (**Figure 1B**). Since NGFR was utilized solely as a reporter gene, the protein level of TCF-1 was further examined by western blot. Intriguingly, it was observed that the protein level of TCF-1 in DT.CAR-T cells was similar to non-transduced T cells. This finding suggests a loss of TCF-1 expression during the generation of CAR-T cells, but it could be restored by the double transduction (**Figure 1C**). We conducted additional experiments using a third-generation retroviral CD33.CAR vector. The results demonstrated TCF-1 also can be overexpressed on CD33.CAR-T cells (**Supplementary Figures S1A–C**).

### Overexpression of TCF-1 improves CAR-T cell expansion by apoptosis resistance

The dynamic of T cell expansion was monitored during the generation phase. Interestingly, CAR-T cells with TCF-1 transduction consistently exhibited a higher cell number starting from day 7 (**Figure 1D**). A downregulation of cleaved caspase 3 and cleaved PARP in DT19.CAR-T cells and non-transduced T cells were observed when compared to CD19.CAR-T cells (**Figures 1E, F**). These findings suggest an increase of active apoptosis in CD19.CAR-T cells, but the upregulation of TCF-1 could reduce the level of active apoptosis. By contrast, DT19.CAR-T cells exhibited a lower percentage and MFI of Ki67 cells compared to CD19.CAR-T cells, while non-transduced T cells showed the lowest percentage and MFI of Ki67 among those three groups (**Figure 1G**). Similar results were also found in CD33.CAR-T and DT33.CAR-T cells (**Supplementary Figures S1E, F**).

### TCF-1 overexpression favors the CD4^+^ component in CAR-T cells and maintains T cell stemness

Previous studies have shown that transcription factor TCF-1 is one of the important regulatory factors in T cell immunity (18–20). TCF-1 can ensure the commitment of CD4^+^ T cells with expression of Th-Pok, while TCF-1 is not essential for CD8^+^ T cells to commit, it still helps CD8^+^ T cells to stabilize (21). Moreover, the proportion of CD4^+^ and CD8^+^ subsets of CAR-T cells is a meaningful effect related with its anti-tumor efficacy (22). Then we verified whether overexpression of TCF-1 would change the CD4/CD8 ratio of CAR-T cells. Our data indicated that there were more CD4^+^ T cells and fewer CD8^+^ T cell in DT19.CAR-T cells than those in CD19. CAR-T cells (**Figure 1H**). Moreover, we also found TCF-1 overexpression upregulated the percentage of T_N_ cells but downregulated the percentage of T_EM_ cells (**Figure 1I**). Similar results were also found in CD33.CAR vector model (**Supplementary Figures S1G, H**).

### Modulation of CAR-T cell surface protein by TCF-1 overexpression

**Figure 2:**
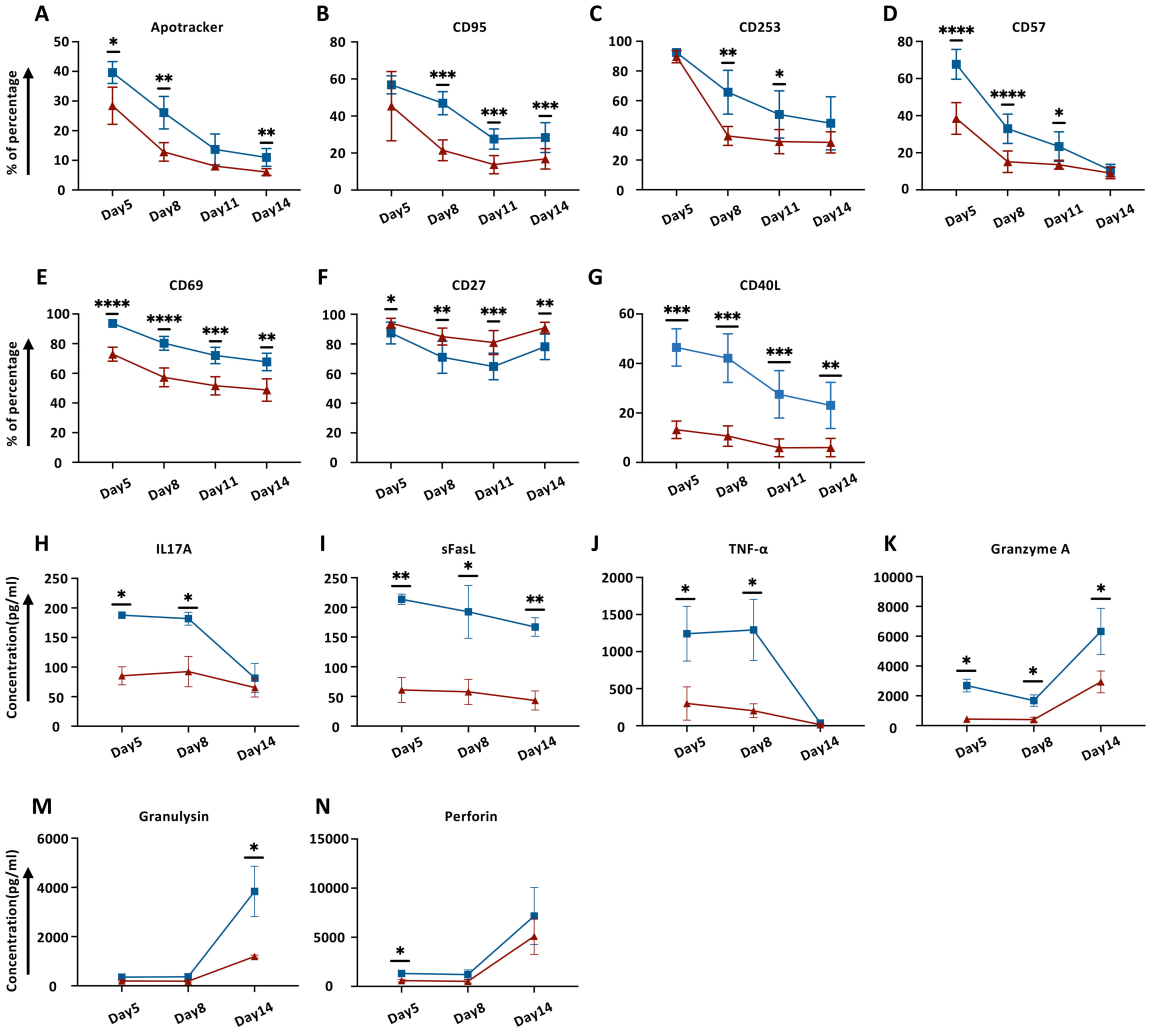
Effect of TCF-1 overexpression on cell surface markers and cytokine release on CD19.CAR-T cells during generation. **(A–G)** Dynamic expression of Apotracker, CD95, CD253, CD57, CD69, CD27, and CD40L on CD19.CAR-T cells and DT19.CAR-T cells at day5, day8, day11, and day14 by flow cytometry (n=6). **(H–N)** Dynamic detection of IL17A, sFasL, TNF-α, Granzyme A, Granulysin, and Perforin on CD19.CAR-T cells and DT19.CAR-T cells from day5, day8, and day 14 by flow cytometry (n=3). A paired t-test was used for statistical analysis. (*P<0.05, **P<0.01, ***P<0.001, ****P<0.0001, ns= no significant difference).

Having successfully generated DT.CAR-T cells, we next examined CAR-T cells and DT.CAR-T cells phenotype during the generation phase. Compared to conventional CAR-T cells, DT.CAR-T cells showed reduced expression of apoptotic markers, such as Apotracker, CD95 and CD253 (**Figures 2A–C**, **Supplementary Figures S2E–G**). Those results indicated TCF-1 overexpression reduced CAR-T cell apoptosis and activation during the generation, which were consistent with the results previously observed in Western Blot.

In parallel, activation markers such as CD57, CD69 and CD40L were markedly upregulated in conventional CAR-T cells (**Figures 2D, E, G**; **Supplementary Figures S2H, I, K**). CD27, which belongs to TNFR superfamily, can enhance CAR-T cell expansion, effector functions, and survival *in vitro*, as well as augmenting CAR-T cell persistence and anti-tumor activity *in vivo* (23). We found that TCF-1 overexpression favored CD27 expression on DT.CAR-T cells (**Figure 2F**, **Supplementary Figure S2J**). Moreover, the co-stimulatory markers, such as CTLA4 and ICOS showed a reduced expression on conventional CAR-T cells at the early stage of generation (**Supplementary Figures S2A, B, M, N**). While CD62L, which is a marker expressed on most of the naïve T cells and central memory T cells (24), was found highly expressed on DT.CAR-T cells at the early stage of generation (**Supplementary Figures S2C, O**).

### Favorable immunoregulatory cytokine profile of CAR-T cell by overexpression of TCF-1

To determine the effect of TCF-1 overexpression on the cytokine profile of CAR-T cells, three different time points were chosen to monitor a broad array of cytokines in the supernatant during the generation. Our results showed that DT.CAR-T cells exerted less potency to secrete CRS-related cytokines such as IL17A, TNF-α, Granzyme A, Granulysin and Perforin. In addition, a steady decrease of souble FasL (sFasL) was observed in the supernatant of DT.CAR-T cells throughout the generation (**Figures 2H–N**).

### Identification of TCF-1 overexpression on diverse CAR-T cell population by machine learning during generation phase

**Figure 3:**
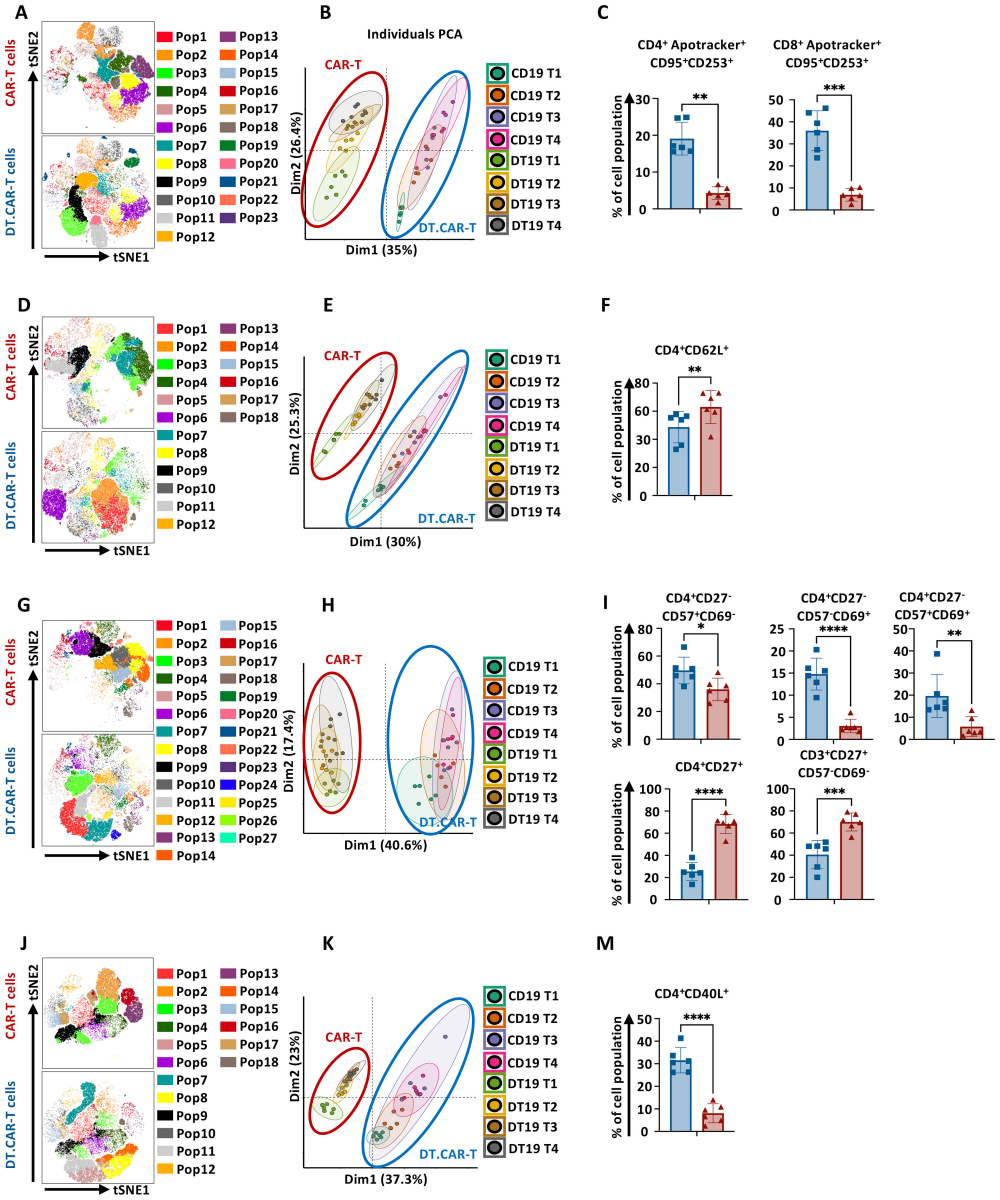
Effect of TCF-1 overexpression on frequency of CD19.CAR-T cell populations. **(A)** t-SNE plot of phonograph identified 23 cell clusters in CD19.CAR-T cells and DT19.CAR-T cells with FACS panel 1 (n=6). **(B)** Evaluation of distinguishing capability of cell cluster by PCA with FACS Panel1. **(C)** Statistic analysis of percentage of CD4^+^Apotracker^+^CD95^+^CD253^+^ and CD8^+^Apotracker^+^CD95^+^CD253^+^ in CD19.CAR-T cells and DT19.CAR-T cells. **(D)** t-SNE plot of phonograph identified 18 cell clusters in CD19.CAR-T cells and DT19.CAR-T cells with FACS panel 2 (n=6). **(E)** Evaluation of distinguishing capability of cell cluster by PCA with FACS Panel 2. **(F)** Statistic analysis of percentage of CD4^+^CD62L^+^ in CD19.CAR-T cells and DT19.CAR-T cells. **(G)** t-SNE plot of phonograph identified 27 cell clusters in CD19.CAR-T cells and DT19.CAR-T cells with FACS panel 3 (n=6). **(H)** Evaluation of distinguishing capability of cell cluster by PCA with FACS Panel 3. **(I)** Statistic analysis of percentage of CD4^+^CD27^-^CD57^+^CD69^-^, CD4^+^CD27^-^CD57^-^CD69^+^, CD4^+^CD27^-^CD57^+^CD69^+^, CD4^+^CD27^+^, CD3^+^CD27^+^CD57^-^CD69^-^ in CD19.CAR-T cells and DT19.CAR-T cells. **(J)** t-SNE plot of phonograph identified 18 cell clusters in CD19.CAR-T cells and DT19.CAR-T cells with FACS panel 4 (n=6). **(K)** Evaluation of distinguishing capability of cell cluster by PCA with FACS Panel 4. **(M)** Statistic analysis of percentage of CD4^+^CD40L^+^ in CD19.CAR-T cells and DT19.CAR-T cells. PCA was performed to identify the major principal components (PCs) that contributed at least 70% of the total variance for day11 and day14. The contribution of each variable (cell subset frequency) in accounting for the variability in these principal components was further analyzed. FACS panel 1: CD3, CD4, Apotracker, CD95, CD253, 7AAD; FACS panel 2: CD3, CD4, CD45RA, CCR7, CD62L, CXCR3, 7AAD; FACS panel 3: CD3, CD4, CD8, CD27, CD57, CD69, 7AAD; FACS panel 4: CD3, CD4, CD40L, CTLA4, ICOS, 7AAD. A paired t-test was used for statistical analysis. (*P<0.05, **P<0.01, ***P<0.001, ****P<0.0001, ns= no significant difference).

The influence of TCF-1 overexpression on the frequency of diverse CAR-T cell populations was comprehensively investigated. An advanced data mining strategy based on machine learning was established and applied to our immunophenotyping data (**Supplementary Figure S3A**), which allowed us to detect previously unknown cell populations in an unbiased, data-driven manner without information loss. In total 23 different cell clusters were identified and projected in the tSNE space (**Figure 3A**). Notably, CD19.CAR-T cells and DT19.CAR-T cells exhibited distinct patterns in a principal component analysis (PCA) plot (**Figure 3B**), indicating TCF-1 overexpression has a strong impact on CAR-T cells. Considering that the clinical utilization of CAR-T cell products is normally after 10 days *ex vivo* expansion, our subsequent downstream analyses only focused on day11 and day14. PCA was performed again to identify the major principal components (PCs) that contributed at least 70% of the total variance for day 11 and day14 (**Supplementary Figure S3B**, left panel). The contribution of each variable (cell subset frequency) in accounting for the variability in these principal components was further analyzed. Through PCA analysis, 13 and 15 cell populations were identified for day11 and day14 (**Supplementary Figure S3B**, right panel), respectively. The commonality of cell populations between day 11 and day 14 was shown in a Venn diagram in **Supplementary Figure S3C**. These 11 common cell populations were further combined into four distinct cell cluster by a manual classification based on the FACS dot plots (**Supplementary Figure S3D**). The retained cell populations were further validated by a manual gating approach and a robustness check. Cell clusters with a cell count of less than 300 were excluded from the analysis. Consequently, two cell populations were found to be significantly correlated with the effect of TCF-1 overexpression. Following this strategy, we found that DT.CAR-T cells obtained less CD4^+^ and CD8^+^ apoptotic cells (**Figure 3C**). This advanced analysis was also applied to differentiation panel (**Figures 3D–F**), activation panel (**Figures 3H–J**), and co-stimulatory marker panel (**Figures 3K–N**). We observed that DT19.CAR-T cells obtained higher frequency of CD4^+^CD62L^+^ T cells, CD4^+^CD27^+^ T cells, while fewer frequency of CD4^+^CD40L^+^ T cells comparing to CD19.CAR-T cells (**Figures 3F, J, N**). In addition, lower activation markers expression was found on DT19.CAR-T cell (**Figure 3J**). The data mining process was applied to validate, and similar results were also found in CD33.CAR vector model (**Supplementary Figures S4–S10**).

### Short-term cytotoxicity of CAR-T cells are not hampered by overexpression of TCF-1 with moderate cytokine producing capacity

**Figure 4:**
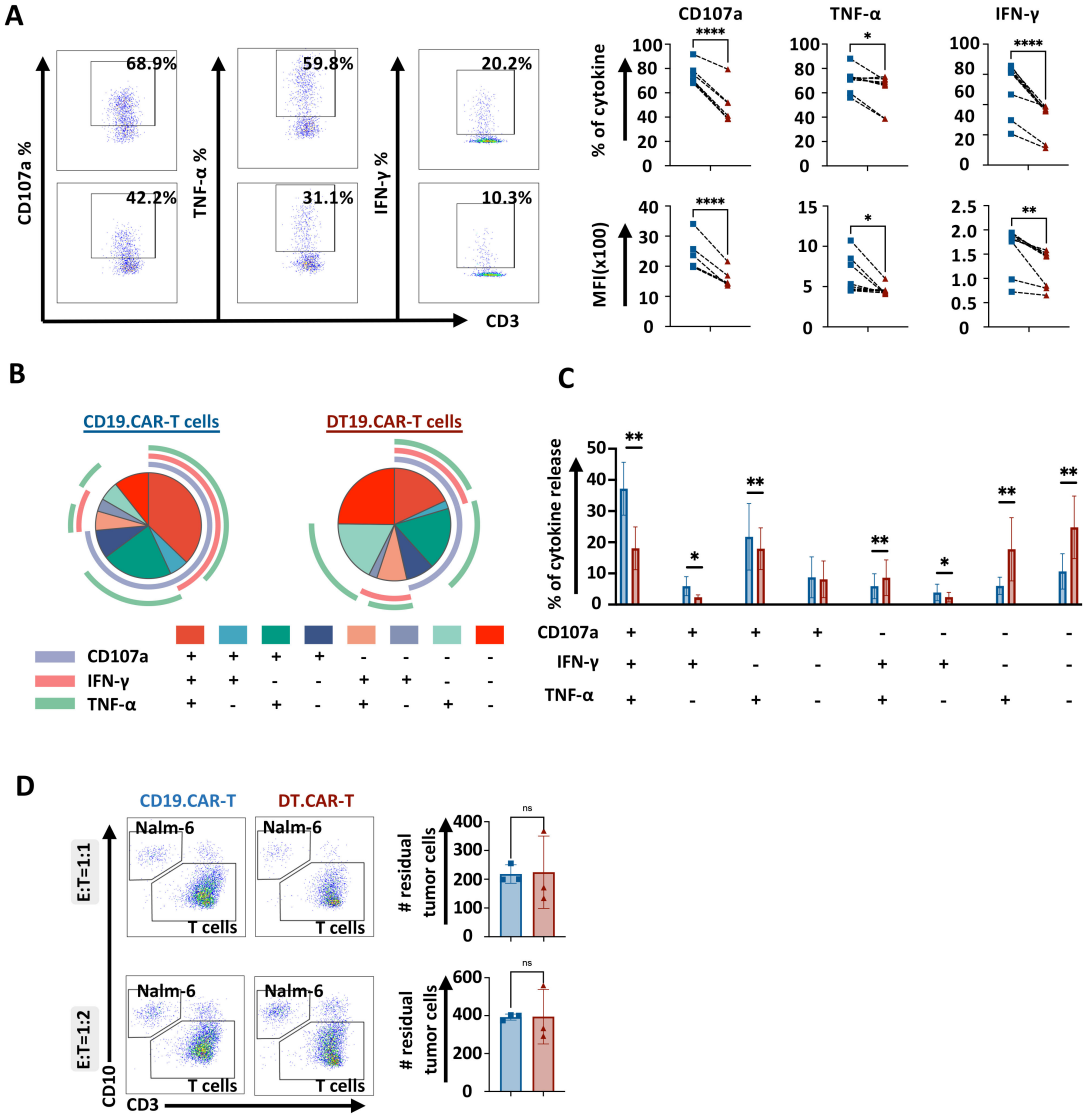
Effect of TCF-1 overexpression on short-term killing efficiency of CD19.CAR-T cells. **(A)** Representative dot plots of percentage of CD107a, TNF-α, IFN-γ on CD19.CAT-T cells and DT19.CAR-T cells. Statistical analysis of percentage and mean fluorescence intensity (MFI) of CD107a, TNF-α, IFN-γ. CD19.CAR-T cells and DT19.CAR-T cells were stimulated by Nalm6 cells for 4 hours. Intracellular cytokine staining was applied to detect the cytokine release. **(B)** Characterization of functional CD19.CAR-T and DT19.CAR-T cell subsets. Based on the expression of CD107a, TNF-α, IFN-γ, T cells can be defined as six functional subsets: CD107a^+^TNF-α^+^IFN-γ^+^, CD107a^+^TNF-α^+^IFN-γ^-^, CD107a^+^TNF-α^-^IFN-γ^+^, CD107a^+^TNF-α^-^IFN-γ^-^, CD107a^-^TNF-α^+^IFN-γ^+^, CD107a^-^TNF-α^+^IFN-γ^-^, CD107a^-^TNF-α^-^IFN-γ^+^, CD107a^-^TNF-α^-^IFN-γ^-^ (n=6). **(C)** Statistic analysis of six functional subsets (n=6). **(D)** Representative dot plots (left) and statistical analysis of killing efficiency of CD19.CAR-T cells and DT19.CAR-T cells after 24 hours in E:T ratio of 1:1 and 1:2 (right). CD19.CAR-T cells and DT19.CAR-T cells were co-cultured with Nalm6 cells respectively for 24 hours, residual tumor cells were detected by flow cytometry after 24 hours.

To determine whether overexpression of TCF-1 would affect the short-term cytotoxic potential and cytokine producing ability of CAR-T cells, conventional CAR-T cells or DT.CAR-T cells were co-cultured with CD19^+^ Nalm6 cells, CD19^+^ Raji cells and CD33^+^ MV4–11 cells, respectively. The expression and MFI of CD107a, TNF-α and IFN-γ were reduced by DT19.CAR-T cells upon Nalm6 stimulation *in vitro* compared to CD19.CAR-T cells (**Figure 4A**). In accordance, TCF-1 overexpression reduced the frequency of multifunctional CAR-T cells in DT19.CAR-T cells (**Figures 4B, C**). Interestingly, the short-term killing efficacy of DT19.CAR-T cells was not affected by overexpression of TCF-1, even though DT19.CAR-T cells showed reduced cytokine release (**Figure 4D**). To confirm our results, different tumor cell lines were applied, and similar results were observed in our study (**Supplementary Figure S11**).

### Superior persistence and long-term killing of CAR-T cells by overexpression of TCF-1 *in vitro*

**Figure 5:**
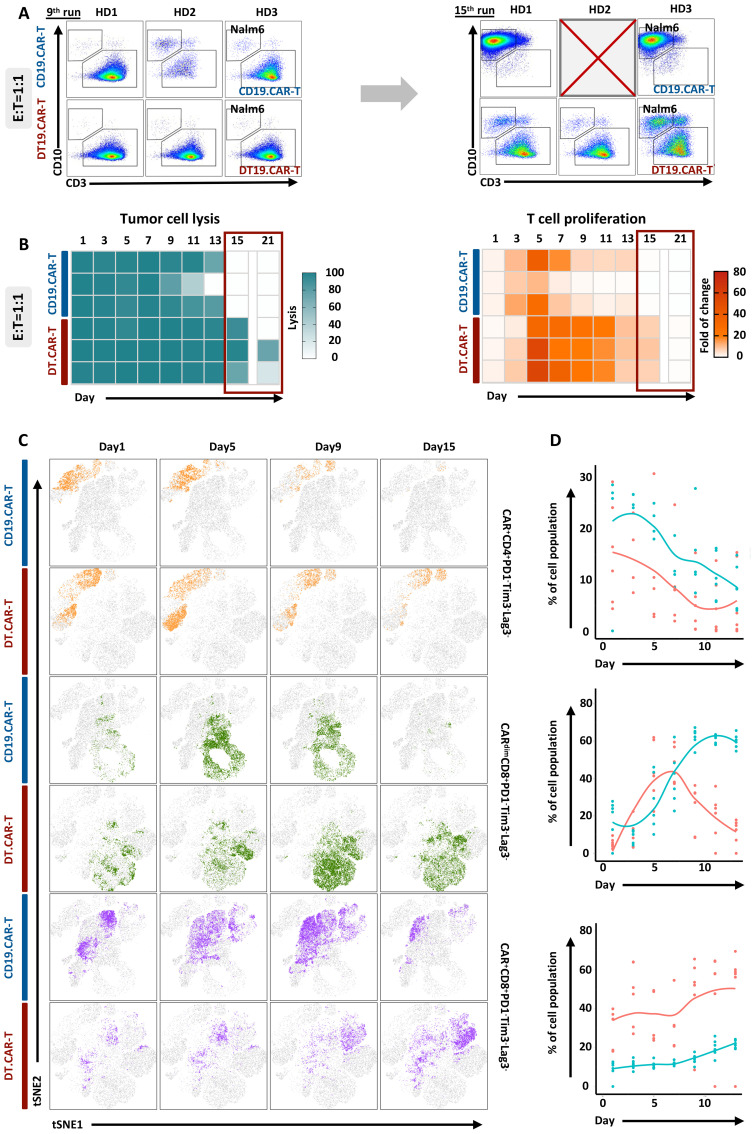
Effect of TCF-1 overexpression on proliferation and long-term cytotoxicity of CD19.CAR-T cells in the co-culture assay. **(A)** Representative dot plots of CD19.CAR-T cell and DT.CAR-T cell proliferation and tumor lysis stimulated by Nalm6 cells after 9 days and 15 days with E: T 1:1, respectively. **(B)** Statistical analysis of Nalm6 cell lysis from day1 to day 21 with E: T 1:1. Darker color (blue) indicates a better tumor lysis, while brighter color (white) indicates a worse tumor lysis. **(C)** Statistical analysis of CD19.CAR-T cell and DT19.CAR-T cell proliferation from day1 to day 21 with E: T 1:1. Darker color (orange) indicates a better cell expansion, while brighter color (white) indicates a worse cell expansion. **(D)** Dynamic changes of cell subsets during co-culture assay. Dynamic change of t-SNE plot of CAR^+^CD4^+^PD1^-^Tim3^-^Lag3^-^, CAR^dim^CD8^+^PD1^-^Tim3^-^Lag3^-^, and CAR^+^CD8^+^PD1^-^Tim3^-^Lag3^-^ cell subsets at day1, day5, day9, and day15 (left panel). Statistical analysis of CAR^+^CD4^+^PD1^-^Tim3^-^Lag3^-^, CAR^dim^CD8^+^PD1^-^Tim3^-^Lag3^-^, and CAR^+^CD8^+^PD1^-^Tim3^-^Lag3^-^ cell subsets from day1 to day12.

To better understand the long-term effect of TCF-1 overexpression on CAR-T cells, the proliferative capacity with the dynamic of cell expansion, the long-term cytotoxicity and the persistence of conventional CAR-T cells and DT.CAR-T cells were investigated as key parameters. Upon continuous stimulation by Nalm6 cells, DT19.CAR-T cells showed a better persistence since day9 (**Figure 5A**, left panel). Moreover, even until day15, there were still T cells persisting in DT19.CAR-T cell model, while there was no T cell left in CD19.CAR-T cells model (**Figure 5A**, right panel).

The long-term anti-tumor activity of CAR-T cells was improved by overexpression of TCF-1, which was evidenced by different number of residual tumor cells upon repetitive tumor challenge (**Figure 5B**, left panel). In addition, we also found that TCF-1 overexpression could extend the proliferation of CAR-T cells (**Figure 5B**, right panel).

### Identification of TCF-1 overexpression on diverse CAR-T cell population by machine learning during co-culture assay

To investigate the interpretability of the clustering results, the protein expression profiles of detected clusters against reference populations was compared. The heatmap displayed median expression intensities for each protein markers, with hierarchical clustering to group rows and columns. Cluster analysis was then used to classify cell subpopulations with similar marker expression according to the level of expression of different markers (**Supplementary Figures S12D, E**). Finally, 10 cell subpopulations were screened (**Supplementary Figure S12F**). Throughout the entire assay, we observed that DT.19 CAR-T cells obtained higher frequency of CAR^+^CD4^+^PD1^-^Tim3^-^Lag3^-^ subpopulation and lower frequency of CAR^+^CD8^+^PD1^-^Tim3^-^Lag3^-^ subpopulation. Interestingly, the CAR^dim^CD8^+^PD1^-^Tim3^-^Lag3^-^ subpopulation increased at the middle-late stage during the co-culture, which indicated CAR density would influence anti-tumor response of CAR-T cells (25). CD33 CAR vector was applied to validate our study and similar results were confirmed (**Supplementary Figures S13, S14**).

### Induction of selective pathways of CAR-T cells by TCF-1 overexpression

**Figure 6:**
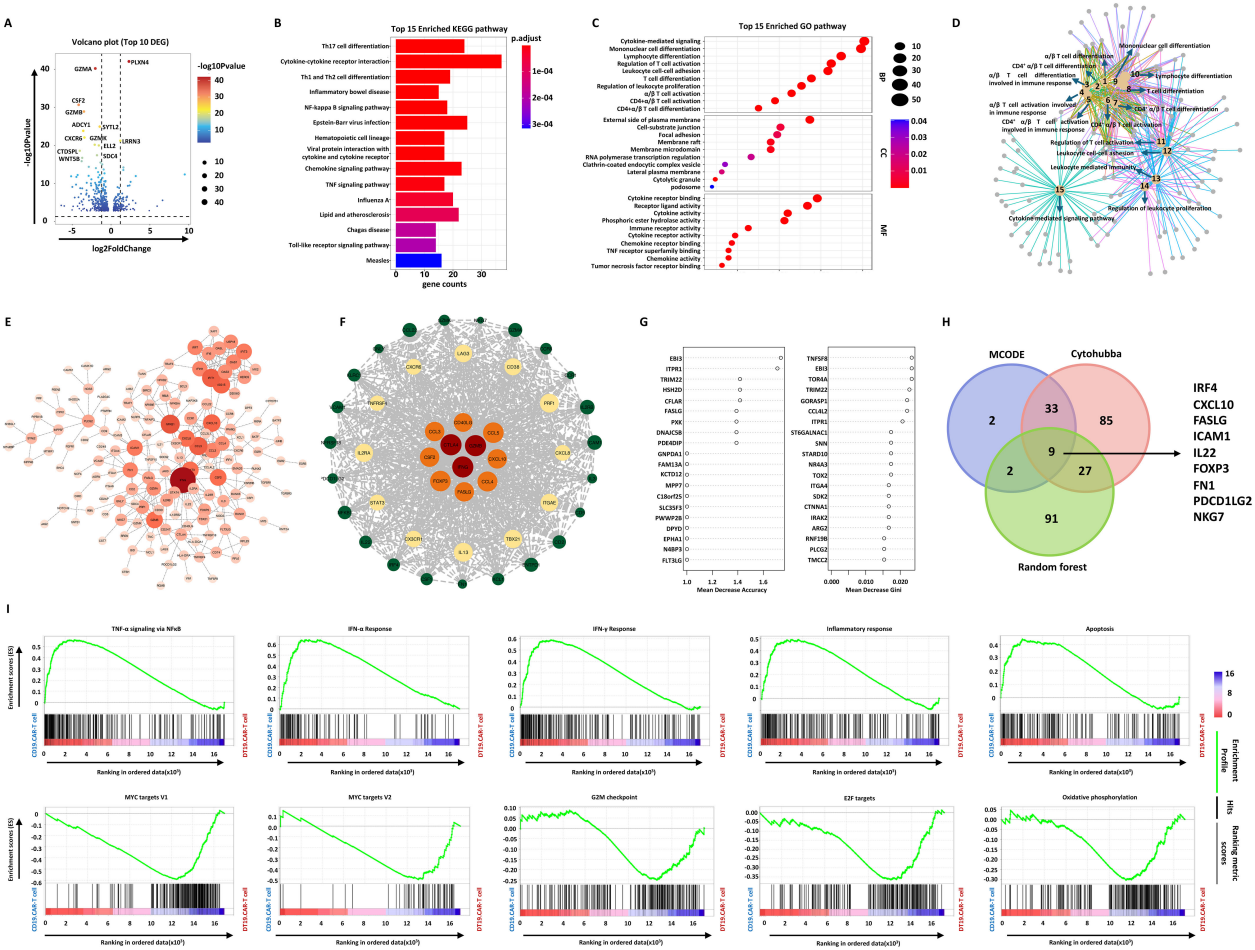
Effect of TCF-1 overexpression on CD19.CAR-T cells at the transcriptional level. **(A)** Volcano map of differentially expressed genes (DEGs) between CD19.CAR-T cells and DT19.CAR-T cells. DEGs were defined as |Log2FC| ≥ 1 and Q value ≤ 0.05. **(B)** KEGG pathway analysis of DEGs, showing the top 15 enriched pathways. The color indicates the *P* adjust value. **(C)** Gene ontology (GO) enrichment analysis of DEGs, showing the top 15 enriched GO terms in biological process, cellular component, and molecular function. The size of nodes is scaled by the gene number. **(D)** Network analysis of enriched cellular component of GO. The size of nodes is scaled by the gene number. **(E)** Protein-protein interaction (PPI) analysis of DEGs by Cytohubba. The top 154 DEGs were colored by orange. **(F)** PPI network analysis of DEGs by MCODE. **(G)** Prioritization of DEGs by random forest analysis. The top 46 DEGs were ranked by mean decrease in accuracy and mean decrease in Gini. **(H)** Identification of hub genes by combination of three different algorithms. The Venn plot illustrates the number of overlap genes in all different algorithms, including Cytohubba, MCODE, and random forest analysis. **(I)** Gene set enrichment analysis of CD19.CAR-T cells and DT19.CAR-T cells. On the x-axis, gene sets represent by vertical black lines, while the enrichment score (ES) is plotted on the y-axis. Points representing genes and their corresponding ES are connected by a green line. The top portion of the plot shows the running ES for the gene set as the analysis walks down the ranked list. The score at the peak of the plot (the score furthest from 0.0) is the ES for the gene set. Gene sets with a distinct peak at the beginning or end of the ranked list are generally the most interesting. The middle portion of the plot shows where the members of the gene set appear in the ranked list of genes. The leading-edge subset of a gene set is the subset of members that contribute most to the ES. For a positive ES, the leading-edge subset is the set of members that appear in the ranked list prior to the peak score. For a negative ES, it is the set of members that appear subsequent to the peak score. The significance threshold is set at false discovery rate (FDR) < 0.05. The color code represents the signal to noise. Samples of three individual donors were tested.

To discovery the mechanism of TCF-1 overexpression on CAR-T cells, we further performed RNA-seq to analyze the final products. The volcano plot showed that TCF-1 overexpression could cause a pronounced alteration of the transcriptional profile of CAR-T cells. In addition, 530 differentially expressed genes (DEGs) were identified when comparing CD19.CAR-T cells to DT19.CAR-T cells (**Figure 6A**). These DEGs were further functionally annotated by Kyoto Encyclopedia of Genes and Genomes (KEGG) (**Figure 6B**) and gene ontology (GO) biological process (**Figure 6C**) enrichment analysis, indicating a strong enrichment in pathways related to cytokine release related pathways, T cell activation and differentiation, especially for CD4^+^ T cells. The relationship of different pathways was also explored by a network analysis (**Figure 6D**), pointing out a similar result as KEGG and GO enrichment analysis.

To screen the hub genes, a protein-protein interaction (PPI) was constructed based on these 530 DEGs, followed by weighting the genes in the network with the Cytohubba plug-in of Cytoscape (**Figure 6E**) and Molecular Complex Detection (MCODE) algorithm (**Figure 6F**). 154 and 46 hub genes were identified by these two algorithms, respectively. The random forest model was applied to rank the DEGs based on the potency to distinguish DT19.CAR-T cells from CD19.CAR-T cells. Mean decrease in accuracy and mean decrease in Gini index determined the ranking of DEGs (**Figure 6G**). The top 20 genes for each index were shown as an example in **Figure 6G**. Incorporating these three different algorithms, 9 downregulated hub genes were identified in our study (**Figure 6H**). Furthermore, an unbiased comparison of CD19.CAR-T cells versus DT19.CAR-T cells was also conducted to identified pathways that might contribute to the TCF-1 overexpression effect. Gene set enrichment analysis (GSEA) was applied to calculate the score for the enrichment of a set of genes in the human molecular signatures database. GSEA identified numerous gene sets enriched in CD19.CAR-T cells that are associated with cytokine release related and apoptosis pathways, while in DT19.CAR-T cells, those genes were enriched in pathways that related to cell cycle, metabolism and DNA repair (**Figure 6I**).

## Discussion

Although CAR-T cell treatment has produced remarkable clinical responses in certain subsets of B-cell leukemia or lymphoma as well as in multiple myeloma, many challenges limit the efficacy of CAR-T cells in hematological malignancies and solid tumors. A major challenge is the persistence of CAR-T cells. There is a need for innovative strategies and approaches for the development of more potent CAR-T cells. TCF-1, an important T- cell regulator, may be a therapeutic option to improve the persistence of CAR-T cells and lead to durable clinical responses.

In this study, we observed that overexpression of TCF-1 reduced apoptosis during CAR-T cell generation and decrease overall T cell activation. This reduction in activation is critical as it leads to decreased activation-induced cell death (AICD), a significant barrier to the persistence and efficacy of CAR-T cell therapies (26). By reducing AICD, TCF-1 overexpression helps to maintain the naïve state of CAR-T cells, which is essential for their long-term persistence and functionality (27). Lower activation levels also correlated with reduced cytokine production, which would be beneficial in preventing toxicities like cytokine release syndrome (CRS). These outcomes are supported by our RNA-seq results that identified the involvement of key hub genes such as IRF4, FOXP3, and FASLG. IRF4 plays a critical role in T cell activation and differentiation, helping to maintain a balance that supports the persistence of less differentiated, stem-like CAR-T cells (28, 29). FOXP3 is associated with regulatory T cells (Tregs) and might contribute to the reduction in activation and cytokine production, suggesting that TCF-1 overexpression could be promoting a regulatory-like phenotype that tempers excessive immune responses (30, 31). FASLG, involved in apoptosis, likely contributes to the observed reduction in cell death, further enhancing CAR-T cell viability (32–34).

Another significant finding in the present study was the prolonged *in vitro* killing efficiency of CAR-T cells overexpressing TCF-1. This enhancement can be attributed to the reduced apoptosis and moderated activation states that preserve the cytotoxic capabilities of CAR-T cells over extended periods. The involvement of hub genes such as CXCL10, ICAM1, and PDCD1LG2 further supports this observation. CXCL10 and ICAM1 are essential for T cell trafficking and adhesion, which are critical for effective tumor localization and infiltration (35–40). Enhanced trafficking and adhesion capabilities suggest that TCF-1 overexpression may improve the ability of CAR-T cells to maintain close contact with tumor cells, thereby prolonging their killing efficiency. Additionally, PDCD1LG2 is associated with T cell exhaustion, and its modulation by TCF-1 indicates that overexpression may delay the onset of exhaustion, thereby extending the functional lifespan of CAR-T cells and enhancing their sustained anti-tumor activity (41–43).

In our experiments, overexpression of TCF-1 also resulted in an increased proportion of CD4^+^ T cells within the CAR-T cell population. CD4^+^ T cells play a critical role in supporting CD8^+^ cytotoxic T cells and orchestrating an effective immune response against tumors (44–46). RNA-seq data revealed that TCF-1 overexpression alters CD4^+^ T cell activation and differentiation, which could influence the balance between various CD4^+^ T cell subsets, such as Th1, Th2, and Tregs. This alteration may contribute to a more effective and sustained immune response, as the proper differentiation of CD4^+^ T cell subsets is crucial for maintaining a balanced and potent anti-tumor environment.

An intriguing aspect of our findings is the observed loss of TCF-1 expression in the third generation of CAR-T cells. This absence suggests that certain elements of the CAR construct might be responsible for downregulating TCF-1 expression. Given the importance of TCF-1 in maintaining CAR-T cell phenotype and function, its loss could contribute to the diminished efficacy and persistence often seen in later generations of CAR-T cells. The restoration of TCF-1 expression through its overexpression in our experiments suggests that the CAR structure itself may interfere with the natural regulation of TCF-1. This interference could be due to the signaling domains within the CAR, which are designed to enhance T cell activation but may inadvertently suppress critical transcription factors like TCF-1 that are necessary for maintaining a less differentiated, stem-like state (47–49).

Understanding the relationship between CAR structure and TCF-1 expression is crucial for the future design of CAR-T therapies. Modifying CAR-T constructs to preserve or enhance TCF-1 expression could improve the overall effectiveness and longevity of CAR-T cells. This insight opens new avenues for the optimization of CAR-T cell constructs, ensuring that key regulatory factors like TCF-1 are maintained to support sustained anti-tumor activity and reduce the risk of exhaustion.

In conclusion, our study highlights the pivotal role of TCF-1 in enhancing CAR-T cell function by reducing apoptosis and activation, prolonging killing efficiency, and modulating CD4^+^ T cell differentiation. The observed loss of TCF-1 expression in third-generation CAR-T cells raises important questions about the impact of CAR construct design on TCF-1 regulation. The rescue of TCF-1 expression through its overexpression suggests potential strategies for optimizing CAR-T cell therapies by preserving essential regulatory pathways. These findings provide a framework for further investigation into the molecular mechanisms governing CAR-T cell efficacy and underscore the importance of transcription factors like TCF-1 in the development of next-generation CAR-T therapies.

## Data Availability

The original contributions presented in the study are publicly available. This data can be found here: https://github.com/PikachuTofu/TCF-1.
