# Supplementary material for: Reshaping CAR-T cells through overexpression of T cell factor 1
Source: Front Immunol. 2025 Nov 10;16:1623869. doi: 10.3389/fimmu.2025.1623869 (PMC12640950; doi:10.3389/fimmu.2025.1623869)
Supplement: Supplementary file 15 [file Table1.docx]

## Materials and methods

## Cell lines

Cell lines (Raji, Nalm6, and MV4-11) were obtained from the German Collection of Microorganisms and Cell Cultures (DSMZ, Braunschweig, Germany) and maintained under the recommended conditions. Moreover, cell lines were periodically authenticated and tested mycoplasma.

## Cell counting

Cell counting with trypan blue and a hemocytometer involves mixing an equal volume of cell suspension and 0.4% trypan blue solution, loading the mixture into a hemocytometer, and counting viable (unstained) and non-viable (blue-stained) cells under a microscope. After cleaning the hemocytometer, 10 µL of the stained cell suspension is loaded into the chamber. Cells in the grid are counted, and the total cell concentration is calculated.

## Western Blot

Protein extractions from the THP-1 cell line were performed using RIPA lysis buffer supplemented with phosphatase and protease inhibitors. The samples were incubated for 20 minutes at 4°C and subsequently stored at −80°C. Prior to Western blot analysis, samples were centrifuged at 16000 xg for 10 minutes. Protein concentrations were determined using the Bradford assay (BioBasic, BDE641). Between 20 and 30 micrograms of protein were loaded onto precast 4-15% gradient Stain-Free gels (4–15% Mini-PROTEAN™ TGX Stain-Free™ Protein Gels, BioRad, 4568086) and electro transferred to Trans-Blot Turbo Mini 0.2 μm nitrocellulose membranes (Bio-Rad, 1704158). Membranes were blocked for 45 minutes at room temperature with 5% milk in PBS-Tween, followed by an overnight incubation at 4°C with the primary antibody in 1% BSA. The subsequent day, the membranes were incubated with the appropriate secondary antibody for 1 hour at room temperature. Protein signals were detected using the ChemiDoc MP Imaging System (Bio-Rad). Densitometric analysis was conducted using ImageLab software (Bio-Rad), with normalization of protein expression to the Stain-Free lane.

## Sorting of CAR-T cells and DT.CAR-T cells

During the extended co-culture assay, CAR-T cells and DT.CAR-T cells were harvested on Day 1, Day 5, and Day 9. Following a washing step with FACS buffer, the cells were stained with CD3 and gt F(ab')2 anti-human IgG antibodies at 4 °C for 30 minutes in the dark. After staining, the cells were washed once with 500 µl of FACS buffer and subsequently resuspended in 1.5 ml of FACS buffer within a 15 ml polypropylene (PP) tube for cell sorting via the BD FACSAria system. Sorting gates were established based on an un-transduced control. The sorted CAR-T cells and DT.CAR-T cells were then collected in 15 ml PP tubes containing 2 ml of PBS supplemented with 2% heat-inactivated fetal bovine serum (h.i. FBS).

## RNA isolation

The sorted CAR-T cells and DT.CAR-T cells were collected via centrifugation at 13,000 rpm for five minutes at room temperature. Subsequently, 350 µl of buffer RLT Plus was added to the resultant cell pellet and vortexed for 30 seconds to ensure complete cell lysis. RNA isolation was then carried out in accordance with the standard protocol provided by the RNeasy® Plus Mini Kit. The concentration of the isolated RNA was determined by measuring the absorbance at 260/280 nm using a Nanodrop spectrophotometer.

## FACS data mining

To comprehensively investigate the overexpression of TCF-1 on the immunophenotype of CAR-T cells and DT.CAR-T cells, two different strategies for data mining have been developed in our current study.

### Cell cluster-based algorithm

To enhance the depth of immunological analysis, our study developed a sophisticated analytical pipeline incorporating advanced flow cytometry (FACS) techniques and machine learning methodologies. A down sampling method was employed to normalize the population sizes across samples. Dimensionality reduction of serial cell populations was achieved through t-distributed stochastic neighborhood embedding (t-SNE). Subsequently, PhenoGraph was utilized to identify cell clusters based on the density of marker expression. The discriminative capacity of these defined cell clusters was assessed using unsupervised analytical methods, including principal component analysis (PCA) and hierarchical clustering. All identified cell clusters were statistically validated, requiring a p-value less than 0.05 when compared to a control group, and further confirmed through manual gating. Cell clusters were ultimately selected based on the clarity and distinctiveness of the cell population morphology.

### Antigen-based algorithm

To investigate the impact of TCF-1 overexpression on antigen expression in CAR-T cells, a semi-automated flow cytometry (FACS) analysis strategy was devised. Initially, dimensionality reduction of the data was conducted using t-distributed stochastic neighborhood embedding (t-SNE). Subsequently, the expression density of each antigen was mapped onto the t-SNE plot. Additionally, the frequency of antigen expression across lineage-specific cell populations was examined and refined through significance analysis.

## Machine learning based advanced analysis

### Unsupervised dimensional reduction

**t-distributed stochastic neighbor embedding (t-SNE).** The Barnes-Hut implementation of t-distributed stochastic neighborhood embedding (t-SNE) is employed as a nonlinear dimensionality reduction tool to decrease the dimensionality of immunophenotyping data, thereby facilitating the simultaneous two-dimensional visualization of expression patterns for all stained markers. This method is particularly effective at preserving local structures within high-dimensional data spaces. The adjustment of the number of iterations, which is crucial for accurately reflecting cellular similarity, was set to 8,000. Additionally, a perplexity value of 30 was chosen to elucidate the distinctions in the immune landscapes across different experimental groups.

**Principal component analysis (PCA).** Principal Component Analysis (PCA) operates as a linear transformation algorithm that facilitates dimensionality reduction to summarize data characteristics, enabling the assessment of similarities and differences among groups, and identifying critical variables. Post-normalization, the frequencies of the cellular subsets, which serve as variables, are scaled to achieve a standard deviation of one and centered to a mean of zero. PCA extracts eigenvalues from the dataset, which quantify the variance retained by each principal component and the contribution of each variable. Analysis is further pursued when the principal components account for at least 70% of the total variance. Subsequently, the contributions of variables to these principal components are scrutinized. Variables that contribute above the expected average are delineated as key factors.

### Unsupervised clustering

**PhenoGraph analysis.** Due to dimensionality reduction, distinct subsets of cells in multidimensional space may visually overlap when projected into lower dimensions. Computational clustering algorithms enable the independent and unbiased identification of cell subpopulations defined by their multidimensional phenotypes. Crucially, these strategies partition cells based on the inherent structure of cellular data, without relying on prior knowledge. PhenoGraph, a robust clustering algorithm, automatically segregates cells into phenotypically distinct subpopulations by constructing a graph that represents phenotypic similarities between cells and subsequently identifying communities within this graph. In our study, PhenoGraph was applied to the dataset, utilizing the input of k-nearest neighbors (k = 30) and the Euclidean distance metric to generate cell clusters.

**Hierarchical clustering.** Cell clusters defined by PhenoGraph were further analyzed using complete linkage clustering, which is based on the similarity of cell cluster proportions. A dendrogram was subsequently constructed to illustrate the relationships between different samples, with branches in the hierarchical tree naturally defining distinct cell clusters.

**LOESS regression.** Locally Weighted Scatterplot Smoothing (LOESS) regression is a non-parametric statistical technique used to estimate underlying trends or relationships in scatterplots. This method is particularly useful for data points exhibiting complex patterns that cannot be adequately captured by simple linear or polynomial regression models. The first step in LOESS regression is the selection of a local neighborhood. For each data point, a subset of nearby data points is selected based on a specified bandwidth or smoothing parameter. Subsequently, weights are assigned to each nearby data point according to their proximity to the central data point, with closer points receiving higher weights and more distant points receiving lower weights. Following this weighting process, polynomial fitting is applied. A low-degree polynomial is fitted to the weighted data points within the local neighborhood, capturing the local trend or relationship among the data points. Finally, the fitted polynomials from different local neighborhoods are combined to form the overall smoothed curve. This combination is achieved by using a weighted average of the local polynomial fits, where the weights are determined by the kernel function and the distance from the central point.

## Gene functional annotation and network analysis

The active interactions among all selected key genes were investigated through protein-protein interaction (PPI) network analysis using the STRING platform. The criteria for defining active interactions included experimental data, database information, co-expression, neighborhood relationships, gene fusion events, co-occurrence, and text mining. The minimum required interaction score was set to medium confidence (0.400). The relationships between genes were visualized as a network map using Cytoscape, excluding genes with no or very few interactions and those distant from the main network.

To further elucidate the functions of genes and gene clusters, genes highly associated with the key markers were functionally annotated using Gene Ontology (GO) term enrichment and Kyoto Encyclopedia of Genes and Genomes (KEGG) pathway enrichment analyses. GO terms and KEGG pathways were deemed significant if both p and q values were below 0.05. Two networks, one for GO terms and the other for KEGG pathways, were constructed using the R package clusterProfiler (version 4.2.2) based on these significant functional annotations. Finally, a pathway enrichment network map was created to illustrate the relationships among the different pathways.

In the exploration of biological processes, hub genes are recognized for their pivotal roles. Utilizing the protein-protein interaction (PPI) network, we identified hub genes by examining network topology characteristics. To facilitate this analysis, Cytoscape software, augmented with the cytoHubba and MCODE plugins, was employed to elucidate key targets and subnetworks within complex biological networks.

Gene set enrichment analysis (GSEA) was employed to identify key pathways and core genes in RNA-seq analysis. This analytical method was utilized to ascertain whether predefined biological processes were significantly enriched in the datasets. Pathways identified as enriched were ranked according to their normalized enrichment scores, with those exhibiting a P < 0.01 selected for further analysis. The GSEA results from various expression profile datasets were compared to determine the common significant KEGG pathways, followed by an in-depth analysis of the core gene sets.

## Quantification and statistical analysis

The statistical analysis for FACS data mining, machine learning based advanced analysis and Gene functional annotation, and network analysis were described previously. A fold of change higher than 1 was defined as the desired size effect. Power calculations were performed with the PROPER package in R and revealed an average marginal power of 0.86 for differential gene expression analysis.

Unless indicated otherwise, all statistical tests comparing two groups were performed using paired t test. *P* < 0.05 were regarded as significant difference. Results are shown as mean ± standard deviation (SD) if not specifically labelled. Plots and statistical tests were mainly performed with GraphPad Prism 9 (San Diego, CA, USA). Figures were carried out with GraphPad Prism 9 (San Diego, CA, USA) and software R (R Foundation for Statistical Computing, Vienna, Austria).

## Supplementary Figures

## Supplementary Figure 1. Effect of TCF-1 overexpression on CAR expression, cell expansion, apoptosis, cell component of CD33.CAR-T cells.

**(A)** Information of CD19 vector, CD33 vector and TCF-1 vector. **(B)** Representative figure of CD33.CAR-T cells and DT33.CAR-T cell generation (n=3). **(C)** Statistical analysis of CD33.CAR and NGFR expression on non-transduced T cells, CD33.CAR-T cells and DT33.CAR-T cells (n=3). **(D)** Statistical analysis protein level of TCF-1 on non-transduced T cells, CD33.CAR- T cells, and DT33.CAR-T cells, as detected by Western Blot (n=3). **(E)** Proliferation dynamics of CD33.CAR-T cells and DT33.CAR-T cells from Day 3 to Day 11 (n=6). **(F)** The expression of protein level of caspase 3, cleaved caspase 3 and PARP in non-transduced T cells, CD33.CAR-T cells and DT33.CAR-T cells (n=3). **(G)** Statistical analysis of the protein level of caspase 3, cleaved caspase3, and PARP in non-transduced T cells, CD33.CAR-T cells and DT33.CAR-T cells (n=3). **(H)** Expression of Ki67 on non-transduced T cells, CD33.CAR-T cells and DT33.CAR-T cells (n=6). **(I)** CD4/CD8 composition in CD33.CAR-T cells and DT33.CAR-T cells (n=6). **(J)** T cell subsets in CD33.CAR-T cells and DT33.CAR-T cells (n=6). A paired t-test was used for statistical analysis. (**P<0.05, **P<0.01, ***P<0.001, ****P<0.0001, ns= no significant difference*).

## Supplementary Figure 2. Effect of TCF-1 overexpression on cell surface markers and cytokine release on CAR-T cells during generation.

**(A-D)** Dynamic expression of CTLA4, ICOS, CD62L, CXCR3 on CD19.CAR-T cells and DT19.CAR-T cells from day5 to day 14 by flow cytometry (n=6). **(E-P)** Dynamic expression of Apotracker, CD95, CD253, CD57, CD69, CD27, CD40L, CTLA4, ICOS, CD62L, and CXCR3 on CD33.CAR-T cells and DT33.CAR-T cells from day5 to day 14 by flow cytometry (n=6).

## Supplementary Figure 3. Workflow of data algorithm.

**(A)** Dimensionality reduction and clustering methods are applied for the advanced flow cytometric analysis. t-distributed stochastic neighbor embedding (t-SNE) is employed to reduce the high dimensional data into a lower-dimensional space, facilitating visualization and exploration of cell populations’ intrinsic structure. Subsequently phonograph clustering is utilized to identify distinct cell populations based on their expression profiles (Panel 1). To further refine the analysis and enable predictive modeling, principal component analysis (PCA) was employed to find the key cell populations retaining the most significant variance. Hierarchical clustering was then implemented to cell populations based on their proximity, allowing to merge the cell populations with a similar marker expression. Finally, the cell populations with statistical significance were reviewed and validated through the manual gating. **(B)** Elbow-shaped scree plot to determine the number of factors to retain in an exploratory principal component (left panel). Contribution of rows to the dimensions (right panel), rows that contribute the most to dimension 1 and dimension 2 or/and dimension 3, in total >70%, are the most important in explaining the variability in the data set. Rows that do not contribute much to any dimension or that contribute to the last dimensions are less important. **(C)** The relationships between most important CD19.CAR-T cell populations at day 11 and day 14 by Venn diagram. **(D)** Manual classification of similar CAR-T cell populations by flowcytometry dot plots. Panel1: CD3, CD4, CAR, NGFR, Apotracker, CD95, CD253, 7AAD. (T3: timepoint 3, day11; T4: timepoint 4, day14; Pop: cell population; C: cell cluster).

## Supplementary Figure 4. Workflow of data mining algorithm CD33.CAR-T cells and DT33.CAR-T cells of FACS panel 1.

**(A)** t-SNE plot of phonograph identified cell clusters of CD33.CAR-T cells and DT33.CAR-T cells. **(B)** Evaluation of distinguishing capability of cell clusters by PCA. **(C)** Dimension reduction by PCA: Elbow-shaped scree plot to determine the number of factors to retain in an exploratory principal component (left panel). Contribution of rows to the dimensions (right panel), Rows that contribute the most to dimension 1 and dimension 2 and dimension 3, in total >70%, are the most important in explaining the variability in the data set. Rows that do not contribute much to any dimension or that contribute to the last dimensions are less important. **(D)** The relationships between most important cell populations at day 11 and day 14 by Venn diagram. **(E)** Manual classification of similar cell populations by flowcytometry dot plots (Pop: cell population, C: cell cluster). **(F)** Statistic analysis of percentage of CD4^+^Apotracker^+^CD95^+^CD253^+^, CD8^+^Apotracker^+^CD95^+^CD253^+^, CD8^+^Apotracker^+^CD95^+^CD253^+^ in CD33.CAR-T cells and DT33.CAR-T cells. Panel1: CD3, CD4, CAR, NGFR, Apotracker, CD95, CD253, 7AAD. (T3: timepoint 3, day11; T4: timepoint 4, day14; Pop: cell population; C: cell cluster).

## Supplementary Figure 5. Workflow of data mining algorithm CD19.CAR-T cells and DT19.CAR-T cells of FACS panel 2.

**(A)** Elbow-shaped scree plot to determine the number of factors to retain in an exploratory principal component (left panel). Contribution of rows to the dimensions (right panel), rows that contribute the most to dimension 1 and dimension 2 or/and dimension 3, in total >70%, are the most important in explaining the variability in the data set. Rows that do not contribute much to any dimension or that contribute to the last dimensions are less important. **(B)** The relationships between most important CD19.CAR-T cell populations at day 11 and day 14 by Venn diagram. **(C)** Manual classification of similar CAR-T cell populations by flowcytometry dot plots. Panel 1: CD3, CD4, CAR, NGFR, CD45RA, CCR7, CD62L, CXCR3, 7AAD. (T3: timepoint 3, day11; T4: timepoint 4, day14; Pop: cell population; C: cell cluster).

## Supplementary Figure 6. Workflow of data mining algorithm CD33.CAR-T cells and DT33.CAR-T cells of FACS panel 2.

**(A)** t-SNE plot of phonograph identified cell clusters of CD33.CAR-T cells and DT33.CAR-T cells. **(B)** Evaluation of distinguishing capability of cell clusters by PCA. **(C)** Dimension reduction by PCA: Elbow-shaped scree plot to determine the number of factors to retain in an exploratory principal component (left panel). Contribution of rows to the dimensions (right panel), Rows that contribute the most to dimension 1 and dimension 2 and dimension 3, in total >70%, are the most important in explaining the variability in the data set. Rows that do not contribute much to any dimension or that contribute to the last dimensions are less important. **(D)** The relationships between most important cell populations at day 11 and day 14 by Venn diagram. **(E)** Manual classification of similar cell populations by flowcytometry dot plots (Pop: cell population, C: cell cluster). **(F)** Statistic analysis of percentage of in CD33.CAR-T cells and DT33.CAR-T cells. Panel 2: CD3, CD4, CAR, NGFR, CD45RA, CCR7, CD62L, CXCR3, 7AAD. (T3: timepoint 3, day11; T4: timepoint 4, day14; Pop: cell population; C: cell cluster).

## Supplementary Figure 7. Workflow of data mining algorithm CD19.CAR-T cells and DT19.CAR-T cells of FACS panel 3.

**(A)** Elbow-shaped scree plot to determine the number of factors to retain in an exploratory principal component (left panel). Contribution of rows to the dimensions (right panel), rows that contribute the most to dimension 1 and dimension 2 or/and dimension 3, in total >70%, are the most important in explaining the variability in the data set. Rows that do not contribute much to any dimension or that contribute to the last dimensions are less important. **(B)** The relationships between most important CD19.CAR-T cell populations at day 11 and day 14 by Venn diagram. **(C)** Manual classification of similar CAR-T cell populations by flowcytometry dot plots. Panel 3: CD3, CD4, CD8, CAR, NGFR, CD27, CD57, CD69, 7AAD. (T3: timepoint 3, day11; T4: timepoint 4, day14; Pop: cell population; C: cell cluster).

## Supplementary Figure 8. Workflow of data mining algorithm CD33.CAR-T cells and DT33.CAR-T cells of FACS panel 3.

**(A)** t-SNE plot of phonograph identified cell clusters of CD33.CAR-T cells and DT33.CAR-T cells. **(B)** Evaluation of distinguishing capability of cell clusters by PCA. **(C)** Dimension reduction by PCA: Elbow-shaped scree plot to determine the number of factors to retain in an exploratory principal component (left panel). Contribution of rows to the dimensions (right panel), Rows that contribute the most to dimension 1 and dimension 2 and dimension 3, in total >70%, are the most important in explaining the variability in the data set. Rows that do not contribute much to any dimension or that contribute to the last dimensions are less important. **(D)** The relationships between most important cell populations at day 11 and day 14 by Venn diagram. **(E)** Manual classification of similar cell populations by flowcytometry dot plots (Pop: cell population, C: cell cluster). **(F)** Statistic analysis of percentage of in CD33.CAR-T cells and DT33.CAR-T cells. Panel 3: CD3, CD4, CD8, CAR, NGFR, CD27, CD57, CD69, 7AAD. (T3: timepoint 3, day11; T4: timepoint 4, day14; Pop: cell population; C: cell cluster).

## Supplementary Figure 9. Workflow of data mining algorithm CD19.CAR-T cells and DT19.CAR-T cells of FACS panel 4.

**(A)** Elbow-shaped scree plot to determine the number of factors to retain in an exploratory principal component (left panel). Contribution of rows to the dimensions (right panel), rows that contribute the most to dimension 1 and dimension 2 or/and dimension 3, in total >70%, are the most important in explaining the variability in the data set. Rows that do not contribute much to any dimension or that contribute to the last dimensions are less important. **(B)** The relationships between most important CD19.CAR-T cell populations at day 11 and day 14 by Venn diagram. **(C)** Manual classification of similar CAR-T cell populations by flowcytometry dot plots. Panel 4: CD3, CD4, CAR, NGFR, CD40L, CTLA4, ICOS, 7AAD. (T3: timepoint 3, day11; T4: timepoint 4, day14; Pop: cell population; C: cell cluster).

## Supplementary Figure 10. Workflow of data mining algorithm CD33.CAR-T cells and DT33.CAR-T cells of FACS panel 4.

**(A)** t-SNE plot of phonograph identified cell clusters of CD33.CAR-T cells and DT33.CAR-T cells. **(B)** Evaluation of distinguishing capability of cell clusters by PCA. **(C)** Dimension reduction by PCA: Elbow-shaped scree plot to determine the number of factors to retain in an exploratory principal component (left panel). Contribution of rows to the dimensions (right panel), Rows that contribute the most to dimension 1 and dimension 2 and dimension 3, in total >70%, are the most important in explaining the variability in the data set. Rows that do not contribute much to any dimension or that contribute to the last dimensions are less important. **(D)** The relationships between most important cell populations at day 11 and day 14 by Venn diagram. **(E)** Manual classification of similar cell populations by flowcytometry dot plots (Pop: cell population, C: cell cluster). **(F)** Statistic analysis of percentage of in CD33.CAR-T cells and DT33.CAR-T cells. Panel 4: CD3, CD4, CAR, NGFR, CD40L, CTLA4, ICOS, 7AAD. (T3: timepoint 3, day11; T4: timepoint 4, day14; Pop: cell population; C: cell cluster).

## Supplementary Figure 11. Effect of TCF-1 overexpression on short-term killing efficiency of CAR-T cells.

**(A)** Statistic analysis of percentage and mean fluorescence intensity (MFI) of CD107a, TNF-α, IFN-γ. CD19.CAR-T cells and DT19.CAR-T cells were stimulated by Raji cells for 4 hours. Intracellular cytokine staining was applied to detect the cytokine release. **(B)** Characterization of functional CD19.CAR-T and DT19.CAR-T cell subsets. Based on the expression of CD107a, TNF-α, IFN-γ, T cells can be defined as six functional subsets: CD107a^+^TNF-α^+^IFN-γ^+^, CD107a^+^TNF-α^+^IFN-γ^-^, CD107a^+^TNF-α^-^IFN-γ^+^, CD107a^+^TNF-α^-^IFN-γ^-^, CD107a^-^TNF-α^+^IFN-γ^+^, CD107a^-^TNF-α^+^IFN-γ^-^, CD107a^-^TNF-α^-^IFN-γ^+^, CD107a^-^TNF-α^-^IFN-γ^-^ (n=9). **(C)** Statistic analysis of six functional subsets (n=6). **(D)** Representative dot plots (left) and statistical analysis of killing efficiency of CD19.CAR-T cells and DT19.CAR-T cells after 24 hours in E:T ratio of 1:1 and 1:2 (right). CD19.CAR-T cells and DT19.CAR-T cells were co-cultured with Raji cells respectively for 24 hours, residual tumor cells were detected by flow cytometry after 24 hours. **(E)** Statistic analysis of percentage and mean fluorescence intensity (MFI) of CD107a, TNF-α, IFN-γ. CD33.CAR-T cells and DT33.CAR-T cells were stimulated by MV4-11 cells for 4 hours. Intracellular cytokine staining was applied to detect the cytokine release. **(B)** Characterization of functional CD33.CAR-T and DT33.CAR-T cell subsets. Based on the expression of CD107a, TNF-α, IFN-γ, T cells can be defined as six functional subsets: CD107a^+^TNF-α^+^IFN-γ^+^, CD107a^+^TNF-α^+^IFN-γ^-^, CD107a^+^TNF-α^-^IFN-γ^+^, CD107a^+^TNF-α^-^IFN-γ^-^, CD107a^-^TNF-α^+^IFN-γ^+^, CD107a^-^TNF-α^+^IFN-γ^-^, CD107a^-^TNF-α^-^IFN-γ^+^, CD107a^-^TNF-α^-^IFN-γ^-^ (n=6). **(C)** Statistic analysis of six functional subsets (n=6). **(D)** Representative dot plots (left) and statistical analysis of killing efficiency of CD33.CAR-T cells and DT33.CAR-T cells after 24 hours in E:T ratio of 1:1 and 1:2 (right). CD33.CAR-T cells and DT33.CAR-T cells were co-cultured with MV4-11 cells respectively for 24 hours, residual tumor cells were detected by flow cytometry after 24 hours.

## Supplementary Figure 12. Effect of TCF-1 overexpression on proliferation and long-term cytotoxicity of CD19.CAR-T cells in the co-culture assay.

**(A)** Representative dot plots of CD19.CAR-T cell and DT19.CAR-T cells proliferation and tumor lysis stimulated by Nalm6 cells after 9 days and 15 days with E: T 1:2, respectively. **(B)** Statistical analysis of Nalm6 cell lysis from day1 to day 21 with E: T 1:2. Darker color (blue) indicates a better tumor lysis, while brighter color (white) indicates a worse tumor lysis. **(C)** Statistical analysis of CD19.CAR-T cell and DT19.CAR-T cell proliferation from day1 to day 21 with E: T 1:2. Darker color (orange) indicates a better cell expansion, while brighter color (white) indicates a worse cell expansion. **(D)** Different cell populations were defined by the expression of protein expression profiles. The heatmap displayed median expression intensities for each protein markers, with hierarchical clustering to group rows and columns. The darker red indicates a higher level of expression, and the darker blue indicates a lower level of expression. **(E)** Cluster analysis was used to classify cell subpopulations with similar marker expression according to the level of expression of different markers. **(F)** 10 cell subpopulations were screened by cell clustering.

## Supplementary Figure 13. Effect of TCF-1 overexpression on proliferation and long-term cytotoxicity of CD33.CAR-T cells in the co-culture assay.

**(A)** Representative dot plots of CD33.CAR-T cell and DT33.CAR-T cell proliferation and tumor lysis stimulated by MV4-11 cells after 9 days with E: T 1:1. **(B)** Representative dot plots of CD33.CAR-T cell and DT33.CAR-T cell proliferation and tumor lysis stimulated by MV4-11 cells after 15 days with E: T 1:1. **(C)** Statistical analysis of MV4-11 cells lysis from day1 to day 21 with E: T 1:1. Darker color (blue) indicates a better tumor lysis, while brighter color (white) indicates a worse tumor lysis. **(D)** Statistical analysis of CD33.CAR-T cell and DT33.CAR-T cell proliferation from day1 to day 21 with E: T 1:1. Darker color (orange) indicates a better cell expansion, while brighter color (white) indicates a worse cell expansion. **(E)** Representative dot plots of CD33.CAR-T cell and DT33.CAR-T cell proliferation and tumor lysis stimulated by MV4-11 cells after 9 days with E: T 1:2. **(F)** Representative dot plots of CD33.CAR-T cell and DT33.CAR-T cell proliferation and tumor lysis stimulated by MV4-11 cells after 15 days with E: T 1:2. **(G)** Statistical analysis of MV4-11 cells lysis from day1 to day 21 with E: T 1:2. Darker color (blue) indicates a better tumor lysis, while brighter color (white) indicates a worse tumor lysis. **(H)** Statistical analysis of CD33.CAR-T cell and DT33.CAR-T cell proliferation from day1 to day 21 with E: T 1:2. Darker color (orange) indicates a better cell expansion, while brighter color (white) indicates a worse cell expansion.

## Supplementary Figure 14. Effect of TCF-1 overexpression on frequency of CD33.CAR-T cell populations in co-culture assay.

**(A)** Different cell populations were defined by the expression of protein expression profiles. The heatmap displayed median expression intensities for each protein markers, with hierarchical clustering to group rows and columns. The darker red indicates a higher level of expression, and the darker blue indicates a lower level of expression. **(B)** Cluster analysis was used to classify cell subpopulations with similar marker expression according to the level of expression of different markers. **(C)** 10 cell subpopulations were screened by cell clustering. **(D-N)** Dynamic changes of CD33.CAR-T cell and DT33.CAR-T cell subsets during co-culture assay.
